# Contralateral non-simultaneous proximal femoral fractures in patients over 65 years old

**DOI:** 10.1007/s00590-021-02929-x

**Published:** 2021-03-17

**Authors:** Francesco Bosco, Jacopo Vittori, Elena Grosso, Mariapaola Tarello, Stefano Artiaco, Alessandro Massè

**Affiliations:** Department of Orthopaedics and Traumatology, University of Torino, Via Zuretti, 29, 10126 Turin, Italy

**Keywords:** Contralateral hip fracture, Proximal femoral fracture, Risk factor, Re-fracture time

## Abstract

**Purpose:**

Epidemiological and clinical parameters according to the Parker-Palmer Index (PPI) have not been specifically studied as predictors of re-fracture time in patients over 65 years old with contralateral hip fracture. The main purpose of this study was to assess whether these parameters could represent a prognostic factor in this population.

**Methods:**

This retrospective study included all consecutive patients older than 65 years that suffered from a proximal femoral fracture, 31 according to Association for Osteosynthesis/Orthopaedic Trauma Association classification, treated at our unit between Feb 1st 2019 and Feb 1st 2020.

**Results:**

This study enrolled 387 patients. Thirty-seven of them had already incurred a contralateral hip fracture: seven males and 30 females. The median time between the first and second hip fractures was 3.5 years. This study revealed that increasing age (*p* = 0.003), male sex (*p* = 0.029) and a PPI value ≥ 5 between the first and second hip fracture (*p* = 0.015) are risk factors associated with a contralateral hip fracture in the first three years after the first episode. There were no statistically significant differences regarding anti-osteoporotic therapy and the anatomic site of the first hip fracture episode.

**Conclusion:**

The results of the present study suggest that several risk factors have a crucial role in hip re-fracture time in patients over 65 years old.

## Introduction

Proximal femoral fractures are among the main causes of disability in the elderly. A large proportion of the patients who undergo a femoral fracture lose part of their independence in daily activities, becoming bedridden, suffering from prolonged hospitalization, needing long-term care in facilities or requiring a walking aid. Another important factor to consider is the mortality rate in this population: 10% at one month from surgery and up to 35% at the end of the first year [1–3].

A hip fracture is often the initial presentation of underlying osteoporosis and affected patients should be considered at risk of developing other fragility-related fractures, including those of the contralateral hip. According to a recent study by Balasubramanian et al., most of the contralateral fractures occur within a 12-month timespan [4]. There is a marked discrepancy between authors regarding the occurrence rate of contralateral hip fracture, with some reporting an incidence of 2.7% at one year and approximately 7.8% at 8.5 years [5]. Other studies concluded that the cumulative incidence is approximately 9% at one year and 20% after five years [6]. The occurrence of contralateral fractures may be related to the mortality rate in these patients. According to different authors, mortality rates at one and five years were 15.9 and 45.4%, respectively, whereas mortality rates in patients who suffered previously from contralateral hip fracture were 24.1 and 66.5%, respectively, at one and five years since the second event [7, 8]. The fracture site and pattern of the second episode on the contralateral limb appeared to be similar to that of the first episode [9].

Risk factors for contralateral hip fractures have been thoroughly assessed. Juhàsz et al. [10] demonstrated that living in a capital city, hip arthroplasty after the first episode, female sex and older age were all risk factors for a contralateral hip fracture. A meta-analysis by Zhu et al. [11] indicated a higher risk of contralateral hip fracture in patients who were female, living in institutions, visually impaired, living with dementia or Parkinson’s disease or suffering from cardiovascular or respiratory diseases. Identification of high-risk groups is crucial for the development of preventive strategies. Fast mobilization after hip fracture is one of the most important factors affecting mortality after surgery.

In some studies, the PPI has proven to be a valid measurement of hip fracture patient mobility and a predictor of mortality with high inter-test reliability [12, 13]. The index is a measurement of the patient’s mobility indoors, outdoors and during shopping that can be easy to investigate at hospital admission and follow-up controls [14, 15]. Furthermore, Pedersen et al. demonstrated a strong correlation of the Parker-Palmer Index with other functional scores (Barthel-20 and Barthel-100) concerning gait function prediction [16]. Mobility score has not been specifically studied in contralateral hip fracture patients as a predictor of re-fracture time. The aim of this study was to retrospectively evaluate epidemiological and clinical parameters according to Parker-Palmer Index. Furthermore, we defined the temporal role they played in a contralateral episode of hip fracture. This finding that has never been investigated in the literature was examined in a consecutive series of patients treated in our unit for one year.

## Methods

We conducted a retrospective study including 387 consecutive patients over 65 years old. All patients suffered from a proximal femoral fracture, 31 according to Association for Osteosynthesis/Orthopaedic Trauma Association (AO/OTA) classification, treated at our Orthopaedics and Trauma Department between Feb 1st 2019 and Feb 1st 2020. Every radiograph has been evaluated and the fractures have been classified by two independent reviewers to reach concordance about the type of fracture. Any patients for whom the reviewers could not agree on the type of fracture were discarded. We excluded from the study patients with periprosthetic hip fractures, revision hip arthroplasties, simultaneous bilateral hip fractures, polytrauma, oncologic hip fractures, 32, 33 AO/OTA hip fractures in the same period.

Patients have been separated into two groups: Group A (*n* = 350) consisted of patients who suffered from a single hip fracture, group B (*n*  = 37) consisted of patients who suffered from a subsequent contralateral hip fracture. Group B was divided into two subgroups: B1 (*n*  = 18) and B2 (*n*  = 19), where members of group B1 suffered a contralateral hip fracture within three years of the first episode and members of group B2 suffered a contralateral hip fracture after this timepoint. For each patient, we recorded gender, age at the first hip fracture, type of primary fracture and type of surgical procedure for the first hip fracture. We compared the total deaths in our patient pool between February 1st 2019 and May 1st 2020. Also, specific data were collected for group B patients, such as age at the contralateral hip fracture, initial (at first hip fracture) and final (at contralateral hip fracture) PPI, pattern of contralateral hip fracture, type of surgical procedure for the contralateral fracture, time between first and contralateral hip fracture, ongoing pharmacological therapy for osteoporosis, surgical timing at the first event, return to the original residence and comorbidities: Parkinson disease, dementia, Alzheimer Disease, cardiovascular and cerebrovascular disease, low vision, syncope, epilepsy, dizziness, orthostatic hypotension, respiratory disease. A logistic regression was performed for group B to evaluate the relationship between re-fracture time and epidemiological and clinical parameters. All information was collected by the authors using a standard proforma with direct patient examination, clinical chart evaluation and telephone interview.

Statistical analysis was performed using Statistical Product and Service Solutions (SPSS Inc., Chicago, Illinois). Descriptive statistical analysis was performed for all demographic and clinical data across the entire cohort. Mean and standard deviation and maximum and minimum values were calculated for continuous variables. Absolute number and frequency distribution were calculated for categorical variables. Subsequently, an unpaired Student’s *t* test was used to analyze normally distributed, continuous data. The chi-square test was used to analyse categorical data. Data were collected using a Microsoft Excel spreadsheet (version 2016; Microsoft, Redmond, WA). The odds ratio (OR) was considered statistically significant with a *p* value < 0.05.

## Results

A total of 387 proximal femoral fractures were registered at our hospital between Feb 1st 2019 and Feb 1st 2020. In patients with a first hip fracture, there was a female predominance (*n* = 280, 71.14%). The number of patients who had died by May 1st 2020 was 31 (8.85%) for group A and three (8.11%) for group B (Table 1).

**Table 1:** Patient population

Prognostic factors	Total patients	Patients without contralateral hip fracture (A)						Patients with contralateral hip fracture (B)					
	*N*°	*N*°	%	Mean ± SD	Median	Min	Max	*N*°	%	Mean ± SD	Median	Min	Max
Patients	387	350	100					37	100				
*Gender*													
Female	280	249	71.14					30	81.08				
Male	108	101	28.86					7	18.92				
*Age during fracture occurred between February 1st 2019 and February 1st 2020*	387	350	100	83 ± 7	84	66	104	37	100	87 ± 5	89	68	95
< 75 (years)		60	17.14					8	21.62				
76–85 (years)		137	39.14					12	32.43				
86–95 (years)		140	48					17	45 95				
> 96(vears)		13	3.72					0	0				
*Type of first hip fracture*	387	350	100					37	100				
Subcapital		53	15.14					6	16 22				
Midcervical		76	21.71					11	29.73				
Basicervical		32	9.14					5	13.51				
Intertrochanteric		168	48					12	32 43				
Subtrochanteric		21	6					3	8.11				
*Type of surgical procedure for first hip fracture*	387	350	100					37	100				
Cancellous screw		2	0.57					2	5.41				
Sliding hip screw		3	086					2	5 41				
Intramedullary nail		198	52.11					17	35.14				
Partial hip replacement		96	27.43					13	45.95				
Total hip replacement		51	14.57					2	5.41				
*Death at May 1st 2020*	387	350	100					37	100				
Yes		31	8.86					3	8.11				
No		319	91.14					34	91.89				

*N* Number (of patients), % Percentage,* SD* Standard deviation, *Min* minimum, *Max* maximus

Thirty-seven patients among the 387 had already incurred a contralateral hip fracture: seven males and 30 females. The median time between the first and second hip fracture was 3.5 years, ranging from 12 days to 24.67 years (Table 2). Correspondence of anatomic site of contralateral hip fracture was the same as the first hip fracture in 19 cases. All patients from group B were examined for PPI. Before the first hip fracture, the mean value was 8 ± 1, between the first and second hip fracture it was 5 ± 2, and after the second hip fracture it was 3 ± 2. The proportion of patients who received anti-osteoporosis medication before the first hip fracture was lower (8.11%) than the proportion who received this medication between their first and second hip fracture (45.95%). Moreover, the majority of hip fracture patients were only treated using supplementation with calcium and vitamin D. The prescription of bisphosphonates markedly increased between the two fractures but remained low, with one patient under treatment before the first hip fracture and six patients under treatment after the contralateral hip fracture. One patient treated with denosumab and another treated with teriparatide did not change medical therapy between the first and second hip fracture.

**Table 2:** Contralateral hip fracture: epidemiological data

Case	Sex	First hip fracture			Contralateral hip fracture			
		Age	Type of fracture	Surgical procedure	Age	Type of fracture	Surgical procedure	Time to second fracture (days)
1	F	62	Subtrochanteric	Intramedullary nail	87	Midcervical	Partial hip replacement	9012
2	F	57	Midcervical	Cancellous screw	80	Midcervical	Partial hip replacement	8610
3	F	74	Midcervical	Partial hip replacement	94	Midcervical	Partial hip replacement	7005
4	F	62	Midcervical	Cancellous screw	78	Midcervical	Total hip replacement	5657
5	F	73	Subcapital	Partial hip replacement	86	Midcervical	Total hip replacement	4776
6	F	80	Pertrochanteric	Intramedullary nail	93	Pertrochanteric	Intramedullary nail	4471
7	F	78	Pertrochanteric	Intramedullary nail	88	Pertrochanteric	Intramedullary nail	3880
8	F	72	Midcervical	Partial hip replacement	84	Midcervical	Partial hip replacement	4357
9	F	79	Midcervical	Partial hip replacement	88	Pertrochanteric	Intramedullary nail	3484
10	F	72	Midcervical	Total hip replacement	82	Pertrochanteric	Intramedullary nail	3447
11	F	82	Basicervical	Sliding hip screw	91	Pertrochanteric	Intramedullary nail	3287
12	F	82	Pertrochanteric	Intramedullary nail	90	Pertrochanteric	Intramedullary nail	2865
13	F	85	Subcapital	Partial hip replacement	91	Subcapital	Partial hip replacement	2128
14	F	88	Midcervical	Partial hip replacement	94	Pertrochanteric	Intramedullary nail	1972
15	F	84	Midcervical	Total hip replacement	89	Pertrochanteric	Intramedullary nail	1740
16	M	80	Basicervical	Sliding hip screw	84	Pertrochanteric	Intramedullary nail	1490
17	F	87	Pertrochanteric	Intramedullary nail	91	Subcapital	Partial hip replacement	1428
18	F	86	Midcervical	Partial hip replacement	89	Pertrochanteric	Intramedullary nail	1279
19	F	86	Subcapital	Partial hip replacement	89	Subcapital	Partial hip replacement	1300
20	F	86	Pertrochanteric	Intramedullary nail	89	Pertrochanteric	Intramedullary nail	810
21	F	82	Midcervical	Total hip replacement	84	Subtrochanteric	Intramedullary nail	848
22	M	89	Pertrochanteric	Intramedullary nail	90	Basicervical	Partial hip replacement	565
23	F	88	Subcapital	Partial hip replacement	89	Subcapital	Partial hip replacement	559
24	F	84	Midcervical	Partial hip replacement	86	Pertrochanteric	Intramedullary nail	447
25	F	91	Pertrochanteric	Intramedullary nail	93	Subtrochanteric	Intramedullary nail	536
26	F	87	Basicervical	Intramedullary nail	89	Pertrochanteric	Intramedullary nail	747
27	F	90	Basicervical	Partial hip replacement	92	Basicervical	Partial hip replacement	681
28	M	66	Pertrochanteric	Intramedullary nail	68	Midcervical	Total hip replacement	589
29	F	77	Subcapital	Partial hip replacement	78	Pertrochanteric	Intramedullary nail	474
30	M	87	Pertrochanteric	Intramedullary nail	89	Pertrochanteric	Intramedullary nail	559
31	M	92	Pertrochanteric	Intramedullary nail	93	Pertrochanteric	Intramedullary nail	313
32	F	86	Subtrochanteric	Intramedullary nail	86	Subtrochanteric	Intramedullary nail	111
33	F	80	Pertrochanteric	Intramedullary nail	80	Pertrochanteric	Intramedullary nail	144
34	F	89	Basicervical	Intramedullary nail	89	Basicervical	Intramedullary nail	224
35	F	95	Pertrochanteric	Intramedullary nail	95	Pertrochanteric	Intramedullary nail	12
36	M	88	Subtrochanteric	Intramedullary nail	88	Basicervical	Intramedullary nail	105
37	M	88	Subcapital	Partial hip replacement	88	Subcapital	Partial hip replacement	59

*M* Male, *F* Female

Patients with contralateral hip fracture were divided into two subgroups according to re-fracture time: group B1 consisted of patients who suffered contralateral hip fracture within three years of the first episode, whereas group B2 consisted of patients who suffered contralateral hip fracture over three years after the first episode (Table 3). A male predominance (*n* = 6, 33.33%) was found in group B1 when compared with group B2 (*n* = 1, 5.26%). There was a statistically significant difference between the two groups (chi-square: 4.75, OR: 9.00, *p* value: 0.029). The mean age at first hip fracture was 86 ± 7 years for the B1 group and 77 ± 9 years for the B2 group, whereas contralateral hip fracture patients had a mean age of 87 ± 6 years and 88 ± 5 years, respectively. These data were analyzed using a t-test and the comparison showed that there was a statistically significant difference between the averages of the two groups. Patients with intra-capsular fractures, compared to those with extra-capsular fractures, had a delayed re-fracture time, but this is not statistically significant (chi-square: 3.28, OR: 3.50, *p* value: 0.070). Analysis of PPI of the two groups showed that a value ≥ 5 was associated with a greater risk of contralateral hip fracture within three years of the first episode. This result was statistically significant (chi-square: 5.90, OR: 6.25, *p* value: 0.015). There was no significant difference between the percentage of patients treated with anti-osteoporotic therapy between the two groups. These data are shown in Table 3.

**Table 3:** Contralateral hip fracture: time to contralateral hip fracture

Patients with contralateral hip fracture(B) (N°37,100%)		Time to contralateral hip fracture(days) < 3 years (B1) (N° 18, 48.65%)									Time to contralateral hip fracture(days) > 3 years (B2) (N° 19, 51.35%)					
		*N°*	%	Mean ± SD	Median	Min	Max	X^2^	OR	*p* value	*N*°	%	Mean ± SD	Median	Min	Max
Gender	M	6	33.33					4.75	9.00	**0.029**	1	5.26				
	F	12	66.67								18	94.74				
PPI between first and contralateral hip fracture	≥ 5	10	55.56					5.900	6.25	**0.015**	3	15.79				
	< 5	8	44.44								15	78.95				
Age during first hip fracture				86 ± 7	88	66	95						77 ± 9	80	57	88
Age during contralateral hip fracture				87 ± 6	89	68	95						88 ± 5	89	78	94
Type of first hip fracture	Subcapital	3	16.67								3	15.79				
	Midcervical	2	11.11								9	47.37				
	Basicervical	3	16.67								2	10.53				
	Petrochanteric	8	44.44								4	21.05				
	Subtrochanteric	2	11.11								1	5.26				
	Extra-capsular fracture	10	44.44					3.28	3.50	0.070	5	26.32				
	Intra-capsular fracture	8	55.55								14	73.68				
Type of contralateral hip fracture	Subcapital	2	11.11								3	15.79				
	Midcervical	1	5.56								6	31.58				
	Basicervical	4	22.22								0	0				
	Petrochanteric	8	44.44								10	52.63				
	Subtrochanteric	3	16.67								0	0				
Type of surgical procedure for first hip fracture	Cancellous screw	0	0								2	10.53				
	Sliding hip screw	0	0								2	10.53				
	Intramedullary nail	12	66.67								5	26.32				
	Partial hip Replacement	5	27.78								8	42.11				
	Total hip Replacement	1	5.56								2	10.53				
Type of surgical procedure for contralateral hip fracture	Cancellous screw	0	0								0	0				
	Sliding hip screw	0	0								0	0				
	Intramedullary nail	13	72.22								10	52.63				
	Partial hip Replacement	4	22.22								7	36.84				
	Total hip Replacement	1	5.56								2	10.53				
Anti-Osteoporotic drugs before first hip fracture	Yes	2	11.11					0.424	2.25	0.515	1	5.26				
	No	16	88.89								18	94.74				
	Ca-Vit D	2	11.11								1	5.26				
	Bisphosphonates	1	5.56								0	0				
	Denosumab	0	0								0	0				
	Teriparatide	0	0								0	0				
Anti-Osteoporotic drugs between first and contralateral hip fracture	Yes	9	50					0.026	1.11	0.873	10	52.63				
	No	9	50								9	47.37				
	Ca-Vit D	9	50								10	52.63				
	Bisphosphonates	2	11.11								4	21.05				
	Denosumab	0	0								1	5.26				
	Teriparatide	0	0								1	5.26				
Death at May 1st 2020		2	11.11								1	5.26				

*N* Number (of patients), %: percentage, *SD* Standard deviation, *Min* Minimum, *Max* Maximus, χ^2^: Chi-square, *OR* Odds ratio, *M* Male, *F* Female, *PPI* Parker-Palmer Index, *Ca* Calcium, *Vit* *D* vitamin D

Therefore, no statistically significant difference (*p* value > 0.05) in re-fracture timing was found between group B1 and B2 regarding the surgical timing at the first event, return to the original residence and comorbidities. The number of patients who had died by May 1st 2020 was two (11.11%) for the B1 group and one (5.26%) for the B2 group respectively. Nevertheless, mortality rate results related to the clinical and epidemiological parameters were not statistically interpretable because our study had a short time of follow-up.

## Discussion

Analysis of our data showed that the incidence of contralateral femoral fractures was 9.56%. This reflects findings from several previous studies in the literature [5, 17, 18]. The median time between the first and contralateral hip fracture was three years. For this reason, in our study, group B was divided into two subgroups with a cut-off of three years of re-fracture time. Studies from Guy et al. and others report similar rates [6, 9]. In five out of the 37 patients, the contralateral hip fracture occurred within six months (range 12–144 days) after the first event, a period during which gait rehabilitation and functional recovery had not yet been completed. Therefore, the failure to rehabilitate and the second traumatic event might be related.

A statistically significant difference was found between the average age at first hip fracture in each group. Particularly, the average age of patients who had re-fractured within three years was significantly above the average age of those who re-fractured later. This confirms that age not only plays an important role in promoting a contralateral hip fracture, as already stated by several authors and confirmed by Zhu Y et al. [11] in their meta-analysis, but it also represents a risk factor that favors a shorter re-fracture time for contralateral hip fracture.

In our study, most of the patients with a proximal femoral fracture were female (71.14%). This difference was even more marked in patients with contralateral hip fractures (76.67%). This value could be related to a longer life expectancy in female patients. Similarly, Gaumetou et al. [9] confirmed in their study a clear predominance of first and contralateral hip fractures in females. In patients with a contralateral hip fracture within three years there was a statistically significant predominance of the male sex (average age: 86 ± 9) compared to the female sex (average age: 88 ± 5). Therefore, male patients have a shorter re-fracture time in cases of contralateral hip fracture. Additional studies with larger samples of patients could be used to perform a multivariate analysis and assess whether this result can be confirmed.

Contralateral hip fractures were often at the same anatomical site as the first fracture. Previous studies have reported between 66.6 and 80.8% correspondence [7, 9, 19], whereas in our case study a correspondence of 51.35% was found. The main morphological criterion affecting fracture site could be the length of the femoral neck: A short neck under 5 cm may favor the onset of a trochanteric fracture, while a neck longer than 5 cm may be more likely to cause an intra-capsular fracture [9]. In cases of asymmetry, the contralateral hip fracture was more frequently extra-capsular, usually treated with an intramedullary nail, according to Shabat et al. This study found a pronounced decrease in bone mass in patients with extra-capsular fractures [19]. This demonstrates an elongated re-fracture time for patients with medial femoral fractures compared to lateral fractures, but this difference was not statistically significant. We hope that future studies further analyse the role of the anatomic pattern of the first hip fracture episode in the occurrence of a short-term contralateral hip fracture.

An interesting finding was a statistically significant difference in re-fracture time for patients with different PPI values. Dividing patients with contralateral hip fracture according to the re-fracture time, we found that patients with a PPI value ≥ 5 had a significantly shorter re-fracture time compared to those with a lower PPI value. This may be related to the patient’s mobility and therefore to a higher risk of falls. Orthopedic surgeons are often the first to treat these patients after a fragility fracture and, because the incidence continues to rise, they may play a more central role in the care of osteoporotic patients [20]. Early diagnosis of a fragility fracture and intervention with medication for osteoporosis is crucial for the prevention of secondary fractures. Although many anti-osteoporosis medications can reduce the risk of subsequent fractures, most patients with a fragility fracture are not treated. Furthermore, patients who use anti-osteoporosis medications often do not adhere properly to the therapy, compromising treatment effectiveness in postoperative management [21]. Patients are frightened by complications of medical therapy (atypical femoral fractures, osteonecrosis of the jaw, myocardial infarction), despite the low incidence of these complications compared with consequences of non-adherence. The use of anti-osteoporosis medications results in an estimated 5–50% reduction in subsequent fracture risk, therefore failing to take medication after a first hip fracture can contribute to an increase in the incidence of a second fracture. Despite several studies showing the importance of preventing fractures due to fragility and second hip fractures, our data show that anti-osteoporotic drugs do not affect the time of second fracture [10, 20–22]. Similarly, other clinical and epidemiological parameters analyzed such as comorbidities, increased surgical timing during the first event and not returning to the original residence, although correlated in the literature with increased risk of contralateral hip fracture and early mortality [10, 11], appeared not to be related to reduced re-fracture timing. Further studies are necessary to confirm the correlation between the parameters analyzed in this study and the contralateral re-fracture timing. Moreover, it may be useful to start studies evaluating biochemical factors already related in the literature with bone healing [23, 24] to investigate their possible role in contralateral hip fracture occurrence and re-fracture timing.

Our study has some limitations. The design of the study is retrospective and the sample size was small for powerful statistical analysis. Therefore, a multiple logistic regression analysis was not performed because the number of observations was insufficient. Moreover, the clinical significance of mortality rate results was limited because our study had a short follow-up period. Nevertheless, the clinical series was homogeneous with many consecutive patients enrolled in our study, that were operated on by the same surgical team.

## Conclusion

A multidisciplinary approach to the treatment of contralateral hip fracture should be employed. This study reveals that several risk factors play a crucial role in hip re-fracture time in patients over 65 years old. Older age, male sex and a PPI value ≥ 5 between the first and second hip fracture are risk factors associated with a contralateral hip fracture within three years of the first episode. There were no statistically significant differences in re-fracture time regarding anti-osteoporotic therapy, anatomic site of the first hip fracture episode, increased surgical timing during the first event, type of comorbidities and not returning to the original residence.

## Data Availability

The dataset analyzed in this study is available from the corresponding author on reasonable request.

## Code availability

Statistical analysis was performed using the Statistical Product and Service Solutions (SPSS Inc., Chicago, Illinois).

## References

[1] Bhandari M, Swiontkowski M (2017). Management of acute hip fracture. N Engl J Med.

[2] Vigni GE, Bosco F, Cioffi A, Camarda L (2021). Mortality risk assessment at the admission in patient with proximal femur fractures: electrolytes and renal function. Geriatr Orthop Surg Rehabil.

[3] Kesmezacar H, Ayhan E, Unlu MC, Seker A, Karaca S (2010). Predictors of mortality in elderly patients with an intertrochanteric or a femoral neck fracture. J Trauma: Injury, Infection, Crit Care.

[4] Balasubramanian A, Zhang J, Chen L, Wenkert D, Daigle SG, Grauer A (2019). Risk of subsequent fracture after prior fracture among older women. Osteoporos Int.

[5] Lawrence TM, Wenn R, Boulton CT, Moran CG (2010). Age-specific incidence of first and second fractures of the hip. J Bone Joint Surg Br.

[6] Ryg J, Rejnmark L, Overgaard S, Brixen K, Vestergaard P (2009). Hip fracture patients at risk of second hip fracture: a nationwide population-based cohort study of 169,145 cases during 1977–2001. J Bone Miner Res.

[7] Sawalha S, Parker MJ (2012). Characteristics and outcome in patients sustaining a second contralateral fracture of the hip. J Bone Joint Surg Br.

[8] Berry SD, Samelson EJ, Hannan MT, McLean RR, Lu M, Cupples LA (2007). Second hip fracture in older men and women: the Framingham Study. Arch Intern Med.

[9] Gaumetou E, Zilber S, Hernigou P (2011). Non-simultaneous bilateral hip fracture: epidemiologic study of 241 hip fractures. Orthop Traumatol Surg Res.

[10] Juhász K, Boncz I, Patczai B, Mintál T, Sebestyén A (2016). Risk factors for contralateral hip fractures following femoral neck fractures in elderly: analysis of the Hungarian nationwide health insurance database. Eklem Hastalik Cerrahisi.

[11] Zhu Y, Chen W, Sun T, Zhang Q, Cheng J, Zhang Y (2014). Meta-analysis of risk factors for the second hip fracture (SHF) in elderly patients. Arch Gerontol Geriatr.

[12] Kristensen MT, Foss NB, Ekdahl C, Kehlet H (2010). Prefracture functional level evaluated by the new mobility score predicts in-hospital outcome after hip fracture surgery. Acta Orthop.

[13] Viberg B, Kold S, Brink O, Larsen MS, Hare KB, Palm H (2020). Is arthroplasty better than internal fixation for undisplaced femoral neck fracture?A national pragmatic RCT: the SENSE trial. BMJ Open.

[14] Parker MJ, Palmer CR (1993). A new mobility score for predicting mortality after hip fracture. J Bone Joint Surg Br.

[15] Voeten SC, Nijmeijer WS, Vermeer M, Schipper IB, Hegeman JH (2020). DHFA Taskforce study group Validation of the fracture mobility score against the Parker mobility score in hip fracture patients. Injury.

[16] Pedersen TJ, Lauritsen JM (2016). Routine functional assessment for hip fracture patients. Acta Orthop.

[17] Scaglione M, Fabbri L, Di Rollo F, Bianchi MG, Dell'omo D, Guido G (2013). The second hip fracture in osteoporotic patients: not only an orthopaedic matter. Clin Cases Miner Bone Metab.

[18] Guy P, Sobolev B, Sheehan KJ, Kuramoto L, Lefaivre KA (2017). The burden of second hip fractures: provincial surgical hospitalizations over 15 years. Can J Surg.

[19] Shabat S, Gepstein R, Mann G, Kish B, Fredman B, Nyska M (2003). The second hip fracture—an analysis of 84 elderly patients. J Orthop Trauma.

[20] Khan AZ, Rames RD, Miller AN (2018). Clinical management of osteoporotic fractures. Curr Osteoporos Rep.

[21] Balasubramanian A, Tosi LL, Lane JM, Dirschl DR, Ho PR, O'Malley CD (2014). Declining rates of osteoporosis management following fragility fractures in the US, 2000 through 2009. J Bone Joint Surg Am.

[22] Bliuc D, Nguyen ND, Nguyen TV, Eisman JA, Center JR (2013). Compound risk of high mortality following osteoporotic fracture and refracture in elderly women and men. J Bone Miner Res.

[23] Serbest S, Tiftikçi U, Tosun HB, Kısa Ü (2017). The Irisin Hormone Profile and Expression in Human Bone Tissue in the Bone Healing Process in Patients. Med Sci Monit.

[24] Serbest S, Tiftikci U, Tosun HB, Gumustas SA, Uludag A (2016). Is there a relationship between fracture healing and mean platelet volume?. Ther Clin Risk Manag.

